# Molecular Survey of Parasitic Contamination of Frozen Berries

**DOI:** 10.3390/pathogens13100900

**Published:** 2024-10-14

**Authors:** Alessandra Barlaam, Marialoreta Datteo, Stefania Perdonò, Antonella Puccini, Annunziata Giangaspero

**Affiliations:** 1 Department of Science of Agriculture, Food and Environment (DAFNE), University of Foggia, 71121 Foggia, Italy; datteom@gmail.com (M.D.); perdonostefania@gmail.com (S.P.); annunziata.giangaspero@unifg.it (A.G.); 2 Azienda Sanitaria Locale, Via Tratturo Castiglione 14, 71122 Foggia, Italy; antopuccini66@gmail.com

**Keywords:** foodborne parasites, frozen berries, real-time PCR

## Abstract

Berries represent healthy dietary options and contain bioactive compounds associated with a decreased risk of diseases. Despite representing healthy food choices, these products can be contaminated by pathogenic microorganisms, including parasites. Among foodborne parasites, *Giardia duodenalis*, *Cryptosporidium parvum*, *Cyclospora cayetanensis*, *Toxoplasma gondii*, and *Echinococcus multilocularis* are of significant public health importance and have been recently detected in fresh berries in Europe, including Italy. Berries can be purchased fresh or frozen, and it is worrying that even frozen berries could represent a risk for the consumer. In fact, several parasites can resist freezing temperatures and have been responsible for outbreaks of infection. The aim of this study was to investigate the presence of *G. duodenalis*, *C. parvum*, *C. cayetanensis*, *T. gondii*, and *E. multilocularis* in frozen berries with simplex and multiplex real-time PCR protocols. A total of 108 packages of mixed frozen berries were bought from supermarkets located in a south-eastern region of Italy. The samples were tested using two simplex real-time PCR protocols targeting *C. parvum* and *G. duodenalis*, respectively, and a multiplex real-time PCR targeting *C. cayetanensis*, *T. gondii*, and *E. multilocularis*. None of the investigated parasites were detected in the frozen berry samples tested. This research topic is still unexplored and of great current interest. These results represent a first attempt to investigate parasitic contamination of frozen berries sold on the Italian market, but further large-scale surveys are required.

## 1. Introduction

Among fresh products, berries hold particular importance due to their exceptional nutritional characteristics, such as high fiber, vitamin, mineral, and phenol content [1], as well as their beneficial properties. Berries are associated with cancer and cardiovascular disease prevention and have beneficial effects on metabolic disorders [2].

The trend toward healthier eating habits in industrialized countries has led to an increase in berry consumption in the United States and Europe, including Italy [3].

*Giardia duodenalis*, *Cryptosporidium* spp., *Toxoplasma gondii*, *Echinococcus* spp., and *Cyclospora cayetanensis* are among the parasites of greatest concern in food production [4,5]. In fact, in a global ranking of foodborne parasites published in 2014 that includes 24 parasites, the ones investigated in the present study were all listed in the top 10, except for *G. duodenalis*, occupying the 11th place, and *C. cayetanensis*, ranking 13th [4]. In another study, foodborne parasites were rated based on their weights across the various regions of Europe. In the south-western ranking, which, among other countries, included Italy, *Echinococcus* spp., *T. gondii*, and *Cryptosporidium* spp. were included. Although *G. duodenalis* and *C. cayetanensis* were not considered a top priority for this specific area, they were listed in the main European ranking, in which they occupied the 9th and the 22nd place, respectively [5]. Although *C. cayetanensis* did not represent one of the main European concerns, since berries are increasingly being imported from Southern and Central America, where cyclosporiasis is endemic, there is increasing attention towards this parasite in Europe [6,7].

*Giardia duodenalis* cysts, *Cryptosporidium* spp., *T. gondii*, and *C. cayetanensis* oocysts, and Taenidae eggs are the resistant forms of these parasites and can contaminate berries at any stage of the production process: during the pre-harvest phase, during harvest, and in post-harvest processes [8,9]. In the case of raspberries, the resistant forms of the parasites easily adhere to the raspberries’ surfaces because they are covered by fine hairs, or they become trapped in the natural grooves or small cracks of the outer layer [8]. Infections occur when people consume food that contains the infectious stages of these parasites, and the risk is particularly high when consuming fresh produce that undergoes minimal or no processing, e.g., berries [8].

*Giardia duodenalis* and *Cryptosporidium* spp. mainly cause gastrointestinal symptoms in humans and also infect a wide range of animals worldwide. While many cases are mild or asymptomatic in immunocompetent people, chronic or more severe infections can occur in vulnerable individuals [10,11]. Humans are the only confirmed hosts of *C. cayetanensis*, which is most commonly associated with diarrhea, nausea, and abdominal pain [12]. Several outbreaks of cyclosporiasis have been linked to the consumption of contaminated berries imported into the USA from Central and South American countries or with travelers returning from endemic areas [8]. Felids are the only final hosts of *T. gondii* and, as such, they are solely responsible for oocyst dissemination. Although toxoplasmosis often remains asymptomatic in immunocompetent individuals, it can cause serious complications during pregnancy [13]. In the European scenario, *E. multilocularis* seems to be exclusively distributed in Central and Northern Europe. However, recent findings suggest that the parasite may be spreading southward [6,14]. Also, the first confirmed autochthonous case of human alveolar echinococcosis in Italy has further emphasized the importance of this parasite in Southern Europe [15]. Humans acquire the infection by consuming food contaminated with the eggs excreted by the final host, primarily red foxes [16].

These parasites are widespread and have been detected on berries worldwide [17,18], including in Europe [7,19]. In Italy, most recently, berries have been found contaminated with *C. cayetanensis*, as well as *G. duodenalis*, several species of *Cryptosporidium*, and *Entamoeba histolytica* [6,20].

European consumers are increasingly interested in frozen berries because of their longer shelf life compared to the fresh ones. This trend is evident both among consumers, for example, in the use of frozen berries for smoothie preparation, and in the industry, which uses frozen products to produce jams, fruit preparations, etc. [21]. The European market for frozen berries is expected to increase by around 1–2% annually in terms of volume. Between 2018 and 2022, the leading European countries for frozen berry consumption were Germany, France, the UK, and Belgium, followed by Italy and the Netherlands [22].

The most concerning aspect is that frozen berries may also pose a risk to consumers despite low-temperature thermal treatment. In fact, previous investigations and experimental studies have shown that such treatment is insufficient, and some parasites are highly resistant to freezing [23].

Molecular techniques are important due to their sensitivity and specificity. Among the various molecular methods available, such as conventional PCR, real-time PCR (qPCR), nested PCR (nPCR), and loop-mediated isothermal amplification assays (LAMP), real-time PCR is the laboratory standard used for the detection of foodborne parasites [8].

The objective of this study was to investigate the presence of *Giardia duodenalis*, *Cryptosporidium parvum*, *Toxoplasma gondii*, *Echinococcus multilocularis*, and *Cyclospora cayetanensis* in frozen berries purchased in stores located in Southern Italy using molecular tools.

## 2. Materials and Methods

### 2.1. Sampling

Between January and July 2023, 108 packages of frozen berries were purchased from four supermarket chains located in the provinces of Foggia and Barletta-Andria-Trani (Apulia region, Southern Italy). Four samples were purchased each week and came from four different brands (A–D). Each package contained varying quantities and proportions of berries: brand A contained 300 g of blackberries, black and red currants, blueberries, raspberries, and strawberries, while brands B and C contained 300 g and 450 g, respectively, of red and black currants, blackberries, raspberries, and blueberries. Brand D contained 300 g of blackberries, red currants, and blueberries. The origin of the berries was not indicated on the packages; however, they were all packaged in Italian facilities. The packages were transferred in coolers to the Parasitology Laboratory at the Department of Agricultural Sciences, Food, Natural Resources and Engineering (DAFNE) at the University of Foggia and were stored at −20 °C in the laboratory. Washing was performed before the expiration date indicated on the packages.

### 2.2. Washing and DNA Extraction of Frozen Berries

For each sampled package, 50 g of berries were weighed and subjected to a washing process following the protocol described in the Bacteriological Analytical Manual (BAM) 19b of the U.S. Food and Drug Administration [6,20,24]. From the pellet obtained, the DNA extraction was performed using the DNeasy^®^ PowerSoil^®^ Pro Kit (Qiagen, Milan, Italy) following the manufacturer’s instructions with slight modifications [6,20].

### 2.3. Real-Time PCR for Giardia duodenalis

For the detection of *G. duodenalis*, the 108 extracted DNA samples were tested using real-time PCR according to the protocol by Klotz et al. [25]. The target sequence, a fragment of the SSU (small subunit rRNA gene), was amplified using the primers Giardia-127F (5′-CGGACACCGCTGGCAA-3′), GiaR (5′-CTGCGTCACGCTGCTCG-3′), and the probe Giardia-152T (HEX-5′-GCCCGCCCTTGCGCGCACG-3′-BHQ2) [25]. The real-time PCR was performed using the CFX96™ Real-Time PCR Detection System (Bio-Rad, Segrate (MI), Italy) in a final volume of 25 µL, using 12.5 µL of 2× KicqStart probe qPCR ready mix low ROX (Sigma Aldrich, Milan, Italy), 8 µL of water, 1 µL of each primer, 0.5 µL of the probe, and 2 µL of DNA [25].

The PCR conditions were as follows: 95 °C for 10 min, 45 cycles at 95 °C for 15 s and at 60 °C for 30 s, and, finally, 72 °C for 30 s [25].

All the samples were tested in triplicate and each experiment included four positive controls and a negative control (ultrapure water).

### 2.4. Real-Time PCR for Cryptosporidium parvum

The real-time PCR for the detection of *C. parvum* was performed by following the protocol of Temesgen et al. [26]. The primers and probe used amplify a 92-base pair (bp) product from a gene encoding thioredoxin peroxidase: TrxPx328F (5′-AGCAAGAACTATGGTGTACTTCTC-3′), TrxPx419R (5′-ACTTCAGAACGAACAACACCCT-3′), and TrxPx353P (FAM-AGGAAGAAGGTATTGCTCTCAGAGGT-MGBEQ) [26]. The real-time PCR was performed using the CFX96™ Real-Time PCR Detection System (Bio-Rad, Segrate (MI), Italy) in a final volume of 20 µL: 10 µL of 2× KicqStart probe qPCR ready mix low ROX (Sigma Aldrich, Milan, Italy), 5.5 µL of water, 1 µL of each primer, 0.5 µL of probe, and 2 µL of DNA [26]. The PCR conditions used were 95 °C for 3 min followed by 45 cycles at 95 °C for 15 s and at 60 °C for 60 s [26].

Each experiment included all the samples tested in triplicate, two positive controls (DNA extracted from *C. parvum* oocysts), and a negative control (ultrapure water).

### 2.5. Multiplex Real-Time PCR for Cyclospora cayetanensis, Toxoplasma gondii, and Echinococcus multilocularis

For the simultaneous detection of *C. cayetanensis*, *T. gondii*, and *E. multilocularis*, a multiplex real-time PCR protocol was used [27]. The primers CyITS1_TT-F (ATGTTTTAGCATGTGGTGTGGC) and CyITS1_TT-R (GCAGCAACAACAACTCCTCATC) and the CyITS1_TT-P (HEX-TACATACCCGTCCCAACCCTCGA-MGBEQ) probe were used for the detection of *C. cayetanensis*, amplifying a 141 bp product from the ITS-1 region. For the detection of *T. gondii*, a 162 bp fragment from the 529 bp sequence of *T. gondii* was amplified using the primers Tox-9F (AGGAGAGATATCAGGACTGTAG) and Tox-11R (GCGTCGTCTC GTCTAGATCG) and the Tox-TP1 (Cy5-CCGGCTTGGCTGCTTTTCCT-MGBEQ) probe [28]. Finally, to amplify a 77 bp product from the 12S rRNA region of *E. multilocularis*, the primers EmMGB_F (GTGCTGCTYATAAGAGTTTTTG) and EmMGB_R (CTATTAAGTCCTAAACAATACCATA) and the EmMGB_P (FAM-ACAACAATATTCCTATCAATGT-MGBEQ) probe were used [29].

The real-time PCR was performed using the CFX96™ Real-Time PCR Detection System (Bio-Rad, Segrate (MI), Italy) in a final volume of 20 µL, using 10 µL of 2× KiCqStart probe qPCR ready mix low ROX (Sigma Aldrich, Milan, Italy) and 2 µL of template DNA. The primers and probes were added in the concentrations and quantities indicated by Temesgen et al. [27] and the reaction conditions used were as follows: 95 °C for 3 min, followed by 45 cycles at 95 °C for 15 s and at 60 °C for 30 s. Each sample was analyzed in triplicate, and positive controls (positive DNA available in the laboratory from previous projects) and a negative control (ultrapure water) for each parasite were included.

## 3. Results

**Figure 1:**
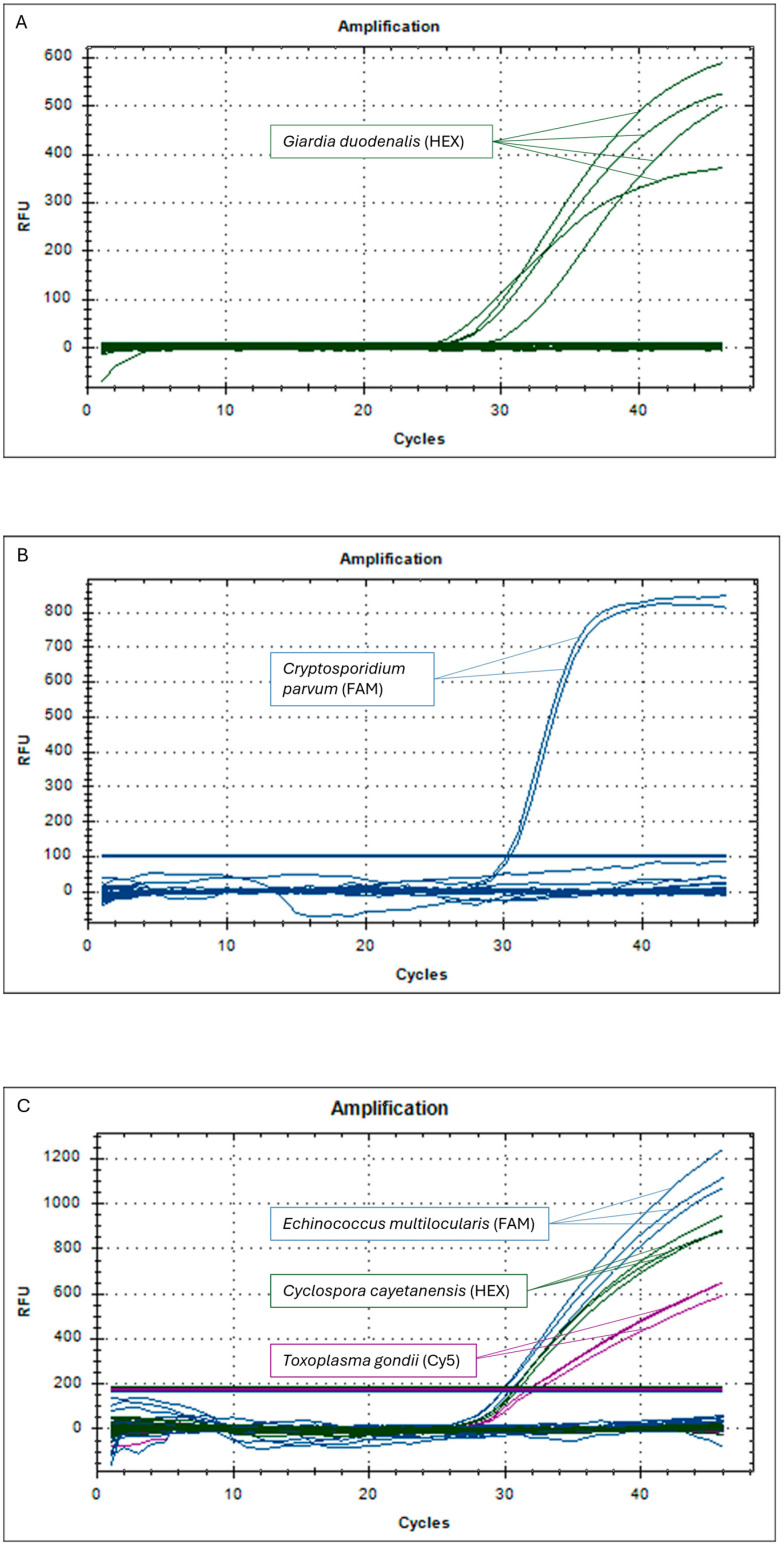
Amplification plot of the simplex real-time PCR for the detection of *G. duodenalis* (**A**) and *C. parvum* (**B**) and the triplex real-time PCR assay for the detection of *C. cayetanensis*, *T. gondii*, and *E. multilocularis* (**C**). RFU = relative fluorescence unit.

The DNA of *G. duodenalis*, *C. parvum*, *C. cayetanensis*, *T. gondii*, and *E. multilocularis* was not detected in any of the 108 samples analyzed. For all the real-time PCR protocols employed in the screening, the positive controls yielded positive results, and the negative controls gave negative results. The results obtained in the present study are shown in Figure 1.

## 4. Discussion

The detection of this study’s target parasites in the analyzed matrices would have been plausible, considering that these parasites have been documented in fresh berries in various European countries, including Italy [6,20] and Norway [7], and that, according to the available literature, several parasites can survive freezing [30,31,32,33,34,35,36,37,38].

*Toxoplasma gondii* oocysts are resistant to freezing, as they survive at −21 °C for 28 days [31,34]. The eggs of *E. multilocularis* also tolerate freezing, surviving at −18 °C for 240 days [30]. Regarding *C. parvum*, a study investigating the infectivity of oocysts suspended in water and stored at various temperatures (−5 °C, −10 °C, −15 °C, −20 °C, and −70 °C) over different time intervals demonstrated that, after storage and biological testing on mice for each treatment type, oocysts stored at −20 °C for up to 8 h maintained viability and infectivity [36]. Conversely, oocysts stored at −20 °C for 24 and 168 h were inactivated by the treatment and were no longer infectious [36]. This study is supported by the findings of Temesgen et al. [35], who evaluated the effectiveness of various treatments, including freezing, on the viability of *Cryptosporidium* oocysts. It was found that the efficacy of inactivation after freezing depended on the exposure time at −20 °C, with longer durations associated with more effective inactivation [35]. Another study evaluated the effect of rapid freezing (blast freezing) on the viability of *C. parvum* oocysts inoculated on green peppers. The freezing process inactivated only 20% of the oocysts; this means that *C. parvum* oocysts are at least partially resistant to rapid freezing [33]. The available data suggest that *Cyclospora* oocysts are also resistant to freezing. In fact, a recent study assessed the effect of freezing raspberries on the recovery of *C. cayetanensis* oocysts using the US-FDA BAM 19b method. Raspberries were spiked with either 200 or 10 oocysts and frozen at −20 °C for 7 days. Freezing the spiked raspberries did not affect the Cq values in either the high or low spike groups [37]. Furthermore, this protozoan was responsible for an outbreak of cyclosporiasis in Philadelphia, USA, in 2000, where raspberries used in a wedding cake filling were the source of the infection despite having been frozen prior to use [32]. It has also been shown that *Trypanosoma cruzi* can survive freezing temperatures in açaí berry pulp. In a study carried out by Bardosa et al. [38], açaí pulp was experimentally contaminated with *Trypanosoma cruzi* and stored at −20 °C for 26 h. After incubation, samples were used to infect mice, and infection rates and parasite virulence were evaluated. However, while freezing at −20 °C reduced the infection rate in mice by 50%, the virulence of the parasites remained unchanged [38]. This indicates that while freezing can decrease the risk of infection, it is not a reliable method for preventing the transmission of Chagas disease through contaminated açaí pulp.

It has been shown that organic matter can protect protozoan parasites from freezing temperatures, as demonstrated by the higher survival rate of *C. parvum* oocysts in mouse feces compared to distilled water [39]. Such findings may be particularly relevant for berry fruits, as the uneven surfaces and the numerous cavities found on the majority of berries may similarly shield oocysts, diminishing the effectiveness of low-temperature treatments [8].

Another aspect to consider is that different approaches have been employed to evaluate the efficiency of low-temperature thermal treatments on different matrices and parasites; therefore, the data available are not always comparable. Lalonde et al. [37] compared contamination levels in berries before and after freezing by analyzing the real-time PCR cycle threshold (Ct) values to evaluate the detectability of *C. cayetanensis* DNA. Real-time PCR, however, only detects the parasites’ DNA and does not reflect any changes in the viability of parasites after freezing. In contrast with this approach, several studies have evaluated the effectiveness of freezing by assessing not only the presence of the investigated parasites but also their viability with a biological test on mice [30,31,36,38], by using propidium iodide and a flow cytometer [33], or, in a more recent approach, by performing an RT-*q*PCR [35].

As for the molecular tools employed in the present study, the DNA extraction kit, the DNeasy^®^ PowerSoil^®^ kit, is currently the preferred method for extracting DNA from oo/cysts and eggs in berries, followed by detection via real-time PCR [26]. Several PCR and real-time PCR protocols for isolating and detecting parasites in fresh products have been described in the literature; however, none are considered a “gold standard” due to the differences in the properties of various food matrices and the parasites of interest. In this study, for each pathogen, we used the most appropriate and efficient molecular protocols based on our previous experience [8]. Although parasitic DNA was not detected in any of the tested samples, all the real-time PCR screening protocols produced positive results for the positive controls, while the negative controls showed no amplification, demonstrating the proper execution and outcome of the reactions (Figure 1).

In this study, the tested samples might have been negative from the source and/or not contaminated along the supply chain due to proper management by the companies involved. Alternatively, they may have been contaminated by such a low number of oocysts and/or eggs that they were undetectable with the washing and real-time PCR protocols employed [25,26,27]. Moreover, the failure to detect the study’s target parasites in the analyzed berry samples could be attributed to the relatively limited number of samples analyzed compared to other broader investigations [6,7,20].

## 5. Conclusions

This research topic is still unexplored and of great current interest; however, the paucity of information available indicates that further studies are required. It is essential to collect data through large-scale investigations on the presence of parasites in fresh and frozen products using standardized analytical methods to obtain unequivocal and comparable results. This approach requires a concerted effort among researchers in the field to cooperate in developing and validating appropriate procedures for detecting parasites in food matrices and would be a starting point for the implementation of HACCP (Hazard Analysis and Critical Control Points) manuals and further preventive measures in the food industry.

## Data Availability

The authors confirm that the data supporting the findings of this study are available within the article.

## References

[1] Zorzi M., Gai F., Medana C., Aigotti R., Morello S., Peiretti P.G. (2020). Bioactive Compounds and Antioxidant Capacity of Small Berries. Foods.

[2] Skrovankova S., Sumczynski D., Mlcek J., Jurikova T., Sochor J. (2015). Bioactive compounds and antioxidant activity in different types of berries. Int. J. Mol. Sci..

[3] Eurostat Fruit and Vegetable Consumption Statistics. https://ec.europa.eu/eurostat/statistics-explained/index.php?title=Fruit_and_vegetable_consumption_statistics&oldid=412723.

[4] Food and Agriculture Organization of the United Nations (FAO)/World Health Organization (WHO) Multicriteria-Based Ranking for Risk Management of Food-Borne Parasites. https://apps.who.int/iris/bitstream/handle/10665/112672/9789241564700_eng.pdf.

[5] Bouwknegt M., Devleesschauwer B., Graham H., Robertson L.J., van der Giessen J.W. (2018). Prioritisation of food-borne parasites in Europe, 2016. Eurosurveillance.

[6] Barlaam A., Temesgen T.T., Tysnes K.R., Rinaldi L., Ferrari N., Sannella A.R., Normanno G., Cacciò S.M., Robertson L.J., Giangaspero A. (2021). Contamination of fresh produce sold on the Italian market with *Cyclospora cayetanensis* and *Echinococcus multilocularis*. Food Microbiol..

[7] Temesgen T.T., Stigum V.M., Robertson L.J. (2022). Surveillance of berries sold on the Norwegian market for parasite contamination using molecular methods. Food Microbiol..

[8] Tefera T., Tysnes K.R., Utaaker K.S., Robertson L.J. (2018). Parasite contamination of berries: Risk, occurrence, and approaches for mitigation. Food Waterborne Parasitol..

[9] Koutsoumanis K., Allende A., Alvarez-Ordóñez A., Bolton D., Bover-Cid S., Chemaly M., Davies R., De Cesare A., Herman L., Hilbert F. (2018). Public health risks associated with food-borne parasites. EFSA J..

[10] Ryan U., Hijjawi N., Feng Y., Xiao L. (2019). *Giardia*: An under-reported foodborne parasite. Int. J. Parasitol..

[11] Zahedi A., Ryan U. (2020). *Cryptosporidium*—An update with an emphasis on foodborne and waterborne transmission. Res. Vet. Sci..

[12] Ortega Y.R., Sanchez R. (2010). Update on *Cyclospora cayetanensis*, a Food-borne and Waterborne Parasite. Clin. Microbiol. Rev..

[13] Hill D.E., Dubey J.P. (2016). *Toxoplasma gondii* as a Parasite in Food: Analysis and Control. Microbiol. Spectr..

[14] Massolo A., Valli D., Wassermann M., Cavallero S., D’Amelio S., Meriggi A., Torretta E., Serafini M., Casulli A., Zambon L. (2018). Unexpected *Echinococcus multilocularis* Infections in Shepherd Dogs and Wolves in South-western Italian Alps: A new endemic area?. Int. J. Parasitol. Parasites Wildl..

[15] Tamarozzi F., Ronzoni N., Degani M., Oliboni E., Tappe D., Gruener B., Gobbi F. (2024). Confirmed Autochthonous Case of Human Alveolar Echinococcosis, Italy, 2023. Emerg. Infect. Dis..

[16] Alvi M.A., Alsayeqh A.F. (2022). Food-borne Zoonotic Echinococcosis: A Review with Special Focus on Epidemiology. Front. Vet. Sci..

[17] Li J., Wang Z., Karim R., Zhang L. (2020). Detection of human intestinal protozoan parasites in vegetables and fruits: A review. Parasites Vectors.

[18] Eslahi A.V., Mamedova S., Nassiba R., Karanis P. (2024). Unveiling risks in healthy food: Vegetables and fruits are linked to the distribution chain of protozoan parasites. Food Microbiol..

[19] Lass A., Szostakowska B., Myjak P., Korzeniewski K. (2015). The first detection of *Echinococcus multilocularis* DNA in environmental fruit, vegetable, and mushroom samples using nested PCR. Parasitol. Res..

[20] Barlaam A., Sannella A.R., Ferrari N., Temesgen T.T., Rinaldi L., Normanno G., Cacciò S.M., Robertson L.J., Giangaspero A. (2022). Ready-to-eat salads and berry fruits purchased in Italy contaminated by *Cryptosporidium* spp., *Giardia duodenalis*, and *Entamoeba histolytica*. Int. J. Food Microbiol..

[21] CBI Market Entry for Frozen Berries. https://www.cbi.eu/market-information/processed-fruit-vegetables-edible-nuts/frozen-berries/market-entry.

[22] CBI Market Potential for Frozen Berries. https://www.cbi.eu/market-information/processed-fruit-vegetables-edible-nuts/frozen-berries/market-potential.

[23] Gérard C., Franssen F., La Carbona S., Monteiro S., Cozma-Petruţ A., Utaaker K.S., Jambrak A.R., Rowan N., Rodríguez-Lazaro D., Nasser A. (2019). Inactivation of parasite transmission stages: Efficacy of treatments on foods of non-animal origin. Trends Food Sci. Technol..

[24] Murphy H.R., Da Silva A.J., Lee S. (2017). Evaluation of an improved U.S. Food and Drug Administration method for the detection of *Cyclospora cayetanensis* in produce using real-time PCR. J. Food Prot..

[25] Klotz C., Radam E., Rausch S., Gosten-Heinrich P., Aebischer T. (2021). Real-Time PCR for molecular detection of zoonotic and non-zoonotic *Giardia* spp. in wild rodents. Microorganisms.

[26] Temesgen T.T., Barlaam A., Tysnes K.R., Robertson L.J. (2020). Comparative evaluation of UNEX-based DNA extraction for molecular detection of *Cyclospora cayetanensis*, *Toxoplasma gondii*, and *Cryptosporidium parvum* as contaminants of berries. Food Microbiol..

[27] Temesgen T.T., Robertson L.J., Tysnes K.R. (2019). A novel multiplex real-time PCR for the detection of *Echinococcus multilocularis*, *Toxoplasma gondii*, and *Cyclospora cayetanensis* on berries. Food Res. Int..

[28] Opsteegh M., Langelaar M., Sprong H., Hartog L.D., De Craeye S., Bokken G., Ajzenberg D., Kijlstra A., van der Giessen J. (2010). Direct detection and genotyping of *Toxoplasma gondii* in meat samples using magnetic capture and PCR. Int. J. Food Microbiol..

[29] Isaksson M., Hagström Å., Armua-Fernandez M.T., Wahlström H., Ågren E.O., Miller A., Holmberg A., Lukacs M., Casulli A., Deplazes P. (2014). A semi-automated magnetic capture probe based DNA extraction and real-time PCR method applied in the Swedish surveillance of *Echinococcus multilocularis* in red fox (*Vulpes vulpes*) faecal samples. Parasites Vectors.

[30] Veit P., Bilger B., Schad V., Schäfer J., Frank W., Lucius R. (1995). Influence of environmental factors on the infectivity of *Echinococcus multilocularis* eggs. Parasitology.

[31] Frenkel J.K., Dubey J.P. (1973). Effects of freezing on the viability of *Toxoplasma* oocysts. J. Parasitol..

[32] Ho A.Y., Lopez A.S., Eberhart M.G., Levenson R., Finkel B.S., da Silva A.J., Roberts J.M., Orlandi P.A., Johnson C.C., Herwaldt B.L. (2002). Outbreak of cyclosporiasis associated with imported raspberries, Philadelphia, Pennsylvania. Emerg. Infect. Dis..

[33] Duhain G., Minnaar A., Buys E. (2012). Effect of chlorine, blanching, freezing, and microwave heating on *Cryptosporidium parvum* viability inoculated on green peppers. J. Food Prot..

[34] Jones J.L., Dubey J.P. (2012). Foodborne toxoplasmosis. Clin. Infect. Dis..

[35] Temesgen T.T., Tysnes K.R., Robertson L.J. (2021). Use of Oxidative Stress Responses to Determine the Efficacy of Inactivation Treatments on *Cryptosporidium* Oocysts. Microorganisms.

[36] Fayer R., Nerad T. (1996). Effects of low temperatures on viability of *Cryptosporidium parvum* oocysts. Appl. Environ. Microbiol..

[37] Lalonde L., Oakley J., Fries P. (2022). Verification and Use of the US-FDA BAM 19b Method for Detection of *Cyclospora cayetanensis* in a Survey of Fresh Produce by CFIA Laboratory. Microorganisms.

[38] Barbosa R.L., Dias V.L., Pereira K.S., Schmidt F.L., Franco R.M.B., Guaraldo A.M.A., Alves D.P., Passos L.A.C. (2012). Survival In Vitro and Virulence of *Trypanosoma cruzi* in Açaí Pulp in Experimental Acute Chagas Disease. J. Food Prot..

[39] Erickson M.C., Ortega Y.R. (2006). Inactivation of Protozoan Parasites in Food, Water, and Environmental Systems. J. Food Prot..

